# Study design

**DOI:** 10.1016/j.mpdhp.2016.06.003

**Published:** 2016-07

**Authors:** Vernon T. Farewell, Daniel M. Farewell

**Affiliations:** **Vernon T Farewell BMath MMath PhD** Programme Leader, MRC Biostatistics Unit, Cambridge Institute of Public Health, Cambridge, UK. Conflicts of interest: None; **Daniel M Farewell MMath PhD** Senior Lecturer in Medical Statistics, Division of Population Medicine, School of Medicine, Cardiff University, Cardiff, UK. Conflicts of interest: None

**Keywords:** clinical trials, diagnostic tests, epidemiology, experimental studies, observational studies, randomization

## Abstract

A brief survey is provided of common designs for medical studies and important issues in their implementation. The designs discussed include those for laboratory studies, clinical trials, cohort studies, case-control and related studies, and diagnostic studies.

## Introduction

The value of a medical study, which we take to be any human health related investigation, depends primarily on the value of the question or questions the study aims to answer and the informativeness of the available data for answering these questions. Both these criteria are strongly influenced by study design. This article aims to provide an overview of the design of medical studies, excluding sample surveys, and of some important issues in planning such studies. There are often overarching considerations of ethics and simplicity that limit what is possible and therefore make it difficult to be prescriptive about what is a good study. Nevertheless, an understanding of general aspects of study design should underlie the planning of any study.

Distinction can be made between clinical and epidemiological studies. The former would often be concerned with the treatment of patients and the latter with “the variation in disease occurrence and the reasons for that variation”.^1^ However, this distinction can become blurred and, in recent years, the field of clinical epidemiology has also emerged, defined by Weiss^2^ as “the study of variation in the outcome of illness and of the reasons for that variation”. In addition, some laboratory studies may not be felt to fall in any of these categories. Another helpful and important distinction is between observational and experimental studies but there are many possible study types that fall between these categories. For the purposes of this article, we will consider only the most common types of studies. For each, we describe the nature of data collection, which will largely make the observational and experimental distinction, and the type of questions being addressed.

The primary designs to be discussed will be for:

1. Laboratory studies
2. Clinical trials
3. Cohort studies
4. Case-control and related studies
5. Diagnostic studies

## Types of outcomes and regression models

### Outcomes

For any study, there is usually a primary outcome, or response, of interest. These may take a variety of forms but common types are:

a. A continuous measurement, e.g. blood pressure, antibody level
b. A yes/no (binary) indicator, e.g. disease versus no disease, relapse versus no relapse
c. The time to an event, e.g. time to death, time to disease
d. A count variable, e.g. number of cells, number of metastases.

### Regression models

For a continuous measurement, it is often assumed that the outcome, Y, follows a normal distribution, perhaps after a transformation of some kind. Analysis will focus on modelling the average or expected value of Y, E(Y), under different conditions. The most common basis is a regression model which assumes that

E(Y) = a + b_1_X_1_ + b_2_X_2_ + … + b_k_X_k_

where the X_i_s represent explanatory variables that code information on study subjects such as treatment or exposure received, age, etc. and the b values are to be estimated and represent the effect of the factor coded by the X variable on Y, all else being equal. In the simple case where (say) X_1_ takes the value 0 for a subject receiving treatment T_1_ and 1 for a subject receiving T_2_, and there are no other explanatory variables, then b_1_ will estimate the difference in E(Y) between a subject receiving T_2_ and one receiving T_1_. If there are other explanatory variables in the model, then b_1_ has the same interpretation but under the additional assumption that all other explanatory variables are the same for the two subjects.

When Y is binary (either 0 or 1), then the regression approach can model the logarithm of the odds of Y = 1 versus Y = 0, [Pr(Y = 1)/Pr(Y = 0)], and, in the case of a binary treatment/exposure indicator X_1_, b_1_ will represent the logarithm of a ratio of the odds of Y = 1 for treatment T_2_ with the odds for treatment T_1_, a so-called *odds ratio*. This methodology is termed logistic regression.

When interest is in a time to event outcome, a regression model will typically be developed for a rate or risk function, r(t), which may depend on time in some fashion. Typically log[r(t)] is modelled and then b_1_ will represent a relative rate function comparing, for example, the rate for subjects receiving T_2_ with those receiving T_1_. This is a comparison, for the two treatments, of the probability of an event occurring at time t given that it has not occurred previously. For more details, see the paper on survival analysis in this issue.^3^

A regression model for a count variable will typically model the logarithm of the average count per unit of time or space which is also a form of rate function. The coefficient b_1_ would then be the logarithm of a relative rate comparing the count rate in subjects receiving T_2_ with those receiving T_1_. The most frequently used model is termed Poisson; the name refers to the distribution assumed for the count variable. Further discussion of these methods can be found in the article on *Regression* in this issue.^4^

### Confounding

Regression models, and related analyses, that allow explanatory variables for more than one factor are particularly valuable in dealing with *confounding*. Confounding arises when an additional factor is related both to an outcome variable and an explanatory factor of primary interest, i.e. treatment or exposure. If this additional factor is not adjusted for in an analysis, often by inclusion in a regression model, then any estimate of the relationship between the outcome and the primary factor may be biased.

## Laboratory studies

The amount of investigator control possible strongly influences study design. Substantial control is often possible in laboratory studies in terms of the number of observations taken and the experimental conditions. An extensive literature on ‘experimental design’ assumes this control and considers quite complex designs. Here, only basic structures are presented to introduce some concepts relevant to many study designs.

For concreteness, assume a study is to compare two treatments, T_1_ and T_2_, and that there is an outcome of interest, e.g. a measure of cell growth, denoted Y. The primary focus of the study will be to compare the expected or average value of Y for cells receiving T_1_ with those receiving T_2_. Additionally, assume that there may be another factor of importance that might influence Y, and, again for concreteness, assume this is a simple two way classification, S_1_ and S_2_, for example, two laboratory technicians.

Table 1 displays two rather idealised study designs, I and II, assuming the use of 100 well test plates. The first plate of design I itself represents a simple design. If interest is restricted to observations in class S_1_, then a comparison of T_1_ and T_2_ could be based on comparing Y values for the 50 wells on Plate 1 receiving T_1_ and the 50 wells receiving T_2_. The precision with which the averages, or expected values, of Y for wells receiving T_1_ and T_2_ can be determined will depend on the number of wells receiving each treatment. This is often termed *replication* and is a central feature of any study design. The observed average values in the two groups will be compared and the replication will provide a measure of the variation in Y against which any difference in the averages can be assessed.

More generally in a study of design I, there will be separate groups of observations in the same class, either S_1_ or S_2_, and within these groups there will be two sets of replicate observations receiving T_1_ and T_2_. Within a class, a comparison of Y values for the two treatments can be made and the information from these comparisons is combined across the different classes to give an overall estimate of how Y depends on the treatment received. The simplest design of this type would be represented by the observations from Plates 1 and 3 of design I. The four rows of design I represent a situation that also allows estimation of the dependence of Y on the S_1_/S_2_ classification. This would be achieved by averaging the Y values for each plate and evaluating the average of these averages in the two rows corresponding to each class in light of the variation of the averages in plates with the same classification.

Design II represents the situation when a study design requires that all subjects in a defined group receive the same treatment. In our example, this might correspond to radiation or no radiation of plates. The comparison of T_1_ and T_2_ must then be made *between* plates. Thus the average value of Y in Plate 1 can be compared with the average value in Plate 2, both being observed in class S_1_, and similarly the average values in Plates 3 and 4 can be compared. The replication in this design comes from having multiple plates for which an average value of Y can be determined.

Table 1 is idealised and the simple averaging of Y values discussed is only generally appropriate with highly ‘balanced’ designs as in Table 1. However, the general principles are applicable to more complicated situations through the use of regression models which also provide suitable measures of variability. A more comprehensive discussion of measures of variability, and how they are reflected in designs such as I and II, is provided in the paper on *Components of Variance* in this issue.^5^

## Clinical trials

### General background

The usual aim of a clinical trial is to evaluate a new treatment for some disease or compare alternative treatments or treatment strategies. Obviously, a good clinical trial should seek to answer an interesting question. The choice of treatments to be compared and the patients on whom they are to be compared largely characterize this. Strict entrance requirements that generate a homogeneous patient population facilitate precise treatment comparisons. However, the use of a more heterogeneous population may be more practically relevant and convincing. The comparison of two highly divergent treatments is simple and likely to produce a result more quickly than a comparison of two similar treatments, or a trial involving more than two treatments. However, more complex designs may allow a more comprehensive set of questions to be addressed. Of course, treatments must also be acceptable to clinicians who must enter their patients into the trial.

Traditionally, clinical trials have been classified in terms of their developmental stage as phase I, II, III or IV; these labels derive primarily from drug development. In this context, phase I trials are primarily dose-finding, phase II studies provide a preliminary investigation of efficacy, phase III designs compare new treatments with standard therapy or no therapy, and phase IV investigations relate to post-marketing surveillance. We focus on comparative trials here.

### Sample size

The size of a study determines the precision with which questions can be answered. This section is relevant to all studies but sample size is particularly important in clinical trials because it also has ethical implications. Formal sample size calculations will typically address one of two design questions:

a. How many subjects are needed?
b. Is the study worth doing if only a specific number of subjects are available?

Such calculations are, it must be remembered, almost always approximate and may not reflect the entire complexity of a design. However, they will be typically based on some primary effect measure such as an estimate of a difference in means, a relative risk or an odds ratio. The variance of this estimated measure will typically be of a form σ^2^/n, where n is the number of subjects in the study and σ^2^ is determined by the particular effect measure of interest and the variability expected in the outcome measure of interest, the latter usually based on prior data and/or publications. Thus precision will increase, i.e. the variance will decrease, as n is increased.

It is often reasonable, and simplest, to determine a planned sample size based on this measure of precision. However, the concept of the power of a study is also widely used. Statistical power is defined as the probability of detecting a designated effect when testing at a specific significance level. Significance testing is discussed in a paper in this issue.^6^ Briefly, a significance test will assess whether there is a non-zero effect of a treatment or exposure on the outcome of interest and will be associated with a particular level of significance, often 5%. This stipulates that an investigator will accept no more than a 5% chance of concluding an effect exists when, in fact, there is no effect, i.e. the false positive rate. Power can be viewed as one minus the false-negative rate for a statistical test.

A power calculation thus requires specification of a significance testing level, together with two of (1) the effect size of medical importance, (2) the power desired and (3) the sample size, the remaining element then being the result of the calculation. The convention, that failure to reject the null hypothesis of no effect is equivalent to concluding that the null hypothesis is true, is inappropriate at the time of analysis but this decision making structure is convenient for power calculations.

It has recently been encouraged, or required, to specify power calculations when reporting study results. One motivation is to consider the magnitude of effect that might have been missed in a ‘negative’ study. However, this is better addressed through confidence intervals that reflect the uncertainty attached to any estimated effect. For example, if an effect of interest lies outside a 95% confidence interval for the estimated effect, then it is unlikely to exist whereas if it is in the interval then it cannot be ruled out. As Cox^7^ writes, power is “quite irrelevant in the actual analysis of data”.

### Treatment assignment

Treatment assignment in a trial is often stratified to guarantee demonstrable balance across one or more known prognostic factors. These factors are used to define separate groups of patients within each of which a balance of treatment assignments is desired. Since this balance ensures these factors are not related to treatment assignment, they cannot confound any estimated treatment effect. However, excessive stratification can be complex and even lead to poor balance if strata are too small. So it is sensible to stratify on only a few major factors. Approximate balance on other factors is maintained through random treatment assignment, crucially also providing protection against *unmeasured* confounding factors.

An alternative to stratification is *minimization*. Minimization avoids the potential problems of stratification with a large number of factors, provided cross-classification of the factors is not thought important. Rather than aiming for balance in each stratum defined by the combination of the prognostic factors, minimization ensures, when each prognostic factor is examined individually, that there is appropriate balance between treatment assignments. Unlike stratification, treatment allocation rules cannot be determined in advance of the study and therefore practical procedures for allocation are more complicated.

When designing a trial, it is increasingly required to specify the statistical procedures intended for the analysis of the trial through a‘statistical analysis plan’. This ensures that investigators think about the primary and secondary outcomes of interest and the specific treatment comparisons of interest. This also avoids a‘search for significance’ by examining many different comparisons based on a variety of outcomes and various subsets of the patient population. However, as argued by Cox and Donnelly,^8^ the trial data, when available, may suggest that additional or alternative analyses could be appropriate and informative. If this leads to a radical change in trial objectives then confirmatory studies will likely be needed. However, as Cox and Donnelly write, “while an initial plan of analysis is highly desirable, keeping at all cost to it alone may well be absurd” because it unduly limits the information available from the study.

### Intention to treat

All patients in a trial should ideally be followed, even if they abandon the treatment protocol. Exclusion of these patients can introduce bias if their failure to complete the protocol is linked to the outcome of treatment. Similarly, the primary analysis should usually compare groups based on originally assigned treatments to assess how they will perform in general use. This is termed an ‘intention to treat’ analysis. It may be of interest to restrict a comparison to those patients receiving and tolerating treatment regimens, addressing the question of ‘the effect of the treatment on the treated’, but the case for such comparisons should be carefully argued (See Matthews and Farewell,^9^ Chapter 18). Note that, in order to avoid bias, treatment assignment should only occur after informed consent procedures. Otherwise, the treatment assigned may influence whether a patient agrees to enter the trial making the treatment groups less comparable.

### Randomization

A randomized clinical trial, in which a patient's treatment is randomly chosen and not therefore predictable, is generally regarded as the best form of evidence. The advantages of a randomized comparison are summarized by Cox^7^ as:

(a) an assurance that in a large experiment it is very unlikely that the estimated treatment effects will be appreciably in error; and
(b) an assurance that the random error of the estimated treatment effects can be measured.

The first assurance, (a), is of primary importance. The fact that a group of patients on one treatment is observed to do better than a corresponding group on an alternative treatment is of value only if it can then be declared that the observed difference is attributable solely to the two treatments and not to something else. Thus, randomization can be seen as the means to establish that treatment *caused* the difference observed. In this regard, approaches such as alternating treatment assignment might be equally effective; however, randomization additionally prevents deliberate or accidental interference in treatment assignments.

A proposed alternative to randomization is the use of ‘historical controls’ from previous studies of the same population of patients. The use of these might be of value in some settings, say in early stage trials, but the arguments for their use are much more complex for comparative trials. Deeks et al.^10^ reviewed this question and concluded:

“Results of non-randomized studies sometimes, but not always, differ from results of randomized studies of the same intervention. Non-randomized studies may still give serious misleading results when treated and control groups appear similar in key prognostic factors. Standard methods of case-mix adjustment do not guarantee removal of bias. …

The inability of case-mix adjustment methods to compensate for selection bias and our inability to identify non-randomized studies that are free of selection bias indicate that non-randomized studies should only be undertaken when randomized controlled trials are infeasible or unethical.”

### Factorial designs

Many clinical trials are designed, primarily, to answer a single question. This may be an unnecessary or even unhelpful restriction. For example, for diseases that require multi-modal therapy, a trial may compare alternative treatments within each mode. This represents a *factorial design* and may make better use of resources or even be essential to appropriately evaluating treatment choices.

### Sequential clinical trials

During a clinical trial, it is common, and often ethically mandated, to prepare interim analyses of accrued data. If one treatment can be shown to be superior, then it is necessary to stop the trial so that all patients may receive the optimal treatment. Unfortunately, the more frequently the study data are examined, the more likely it is that a ‘statistically significant’ result will be observed even if there is no difference between the treatments. For example a single test may have a 5% chance of a false-positive result. However, if five such tests are done, then the chance of at least one of the tests being positive is 14.2%.

This phenomenon has led to specialized techniques to ‘monitor’ clinical trials. Fleming et al.^11^ argue that treatment differences observed in the early stages of a trial may occur for a variety of reasons, and that the primary purpose of a sequential design is to protect against unexpectedly large treatment differences. Therefore, Fleming et al. advocate using group sequential designs based on scheduled repeated analyses that do this but preserve sensitivity to late-occurring survival differences.

### Equivalence trials

A trial to test the equivalent efficacy of two treatments, say because a new treatment is expected to have lower toxicity or cost than a current treatment, cannot be based on a failure to reject a significance test because this does not establish equivalence. Thus, equivalence trials are generally designed to establish that the relative efficacy between two treatments does not exceed some specific, usually clinically unimportant, level.

Essentially, the trial will be designed to provide a confidence interval for the relative efficacy that is small enough to rule out unacceptably large differences. If it is only knowledge that a new treatment is no worse than a standard that is of interest, then the term ‘non-inferiority trial’ is used.

### Other designs

The most common design for a clinical trial is the so-called *parallel group design* where patients are individually randomized. Two more complex designs are *cross-over trials* and *cluster randomized studies*.

Cross-over designs can be used when patients can be treated sequentially with more than one treatment. For example, different symptomatic treatments for asthma could be made available to a patient during successive months. If two treatments, say A and B, are under study and two time periods will be used, then the classic two-period cross-over design would enrol patients and then randomly assign them to receive treatments in the order AB or BA.

The potential advantage of a cross-over design is that treatments can be compared within patients rather than between patients, usually leading to more precise comparisons. However, if the effect of treatments might ‘carry-over’ from one period to the next, then a cross-over design is not recommended. Extensions to more than two periods are possible.

Cluster randomized trials, or group randomized trials, arise when randomization of individual patients is not possible or inconvenient. For example, varying the treatment provided from patient to patient within the same medical centre or practice might be difficult or an educational intervention may be designed for a classroom setting. In such cases, a group of study subjects are effectively randomized together to the same intervention.

In such trials, the analysis must compare treatments at the level of randomization and allow for correlation between outcomes for subjects randomized together, arising because these outcomes will be more similar than those for patients in different clusters. A common approach to the analysis of such trials is to use random effects models. (See paper on components of variance in this issue^5^). It is critical not to ignore the clustering because this design will provide less precise estimates of treatment effects than those available from a comparably sized individually randomized trial.

## Cohort studies

In a cohort study, a population or random sample of a population is monitored longitudinally for a period of time. Important characteristics of each cohort member are ascertained at the start of the study, or when a subject ‘enters’ the cohort, and during the period of follow-up. This information is used to define the explanatory variables or ‘risk factors’ that may be related to outcomes of interest.

A key feature is that potential risk factors are measured prospectively, that is, before the outcome is observed. However, some or all risk factor information, or indeed outcome information, may not necessarily be acquired at the time of measurement. A distinction is therefore sometimes made between retrospective, or historical, cohort studies and prospective cohort studies. A prospective cohort follows each individual and acquires information after the individual agrees to enter the study. In the classical historical cohort study, the period of follow-up has usually occurred before the study is undertaken. Essentially the study is a reconstruction of past events. This may be cheaper and faster than a prospective cohort study but depends on having accurate exposure data on subjects during the past, avoiding issues such as recall bias, and accurate follow-up data on virtually all subjects. Of course, there can be a range of variation between these two extremes when some information is acquired retrospectively and other prospectively. However, once appropriate data are acquired, by whatever means, the analysis would proceed as if the data were acquired prospectively.

### Epidemiological cohort studies

The cohort study is a primary tool for the study of disease incidence, importantly providing a direct estimate of the rate of disease incidence in population subgroups defined by the explanatory variables.

As well as through the prospective/retrospective data collection distinction, epidemiological cohort studies can also be distinguished by whether they have an internal or external control group who do not experience an exposure of interest. An internal control group arises when study subjects include unexposed individuals. If such a group is not present, for example in a study of workers with a common occupational exposure, then study subjects may be compared with an external standard. This might be derived from national mortality or morbidity data, but may not be appropriate for all purposes. For example, the so-called ‘healthy worker effect’ suggests that employed individuals will be, on average, healthier than the general working age population and thus there is a bias against detecting adverse conditions when comparing an occupational cohort to national age-specific data. Comparisons within the cohort are generally to be preferred and an important design criterion is to ensure there is sufficient detail on individual exposures to allow detailed analysis of differential exposure levels.

Prospective epidemiological cohort studies are subject to the limitation that the information collected is determined when the study is initiated. Typically there will be a few major outcomes that will be monitored and the explanatory variable information will reflect what is known about possible disease risks. During the course of the study, other information may suggest other outcomes or other risk factors might be of interest. It is generally difficult to compensate for this by additional retrospective data collection.

### Clinical cohort studies

A clinical cohort typically derives from longitudinal follow-up of patients with a specific disease. Much more commonly than in epidemiological cohorts, patients will enter the cohort over a considerable period of time, the recruitment period. The basic data structure is the same as that for an epidemiological cohort but the outcomes of interest will now typically be disease related outcomes. Also, the risk factors of interest will generally be based on clinical/laboratory/genetic information as well as demographic factors. For example, based on a clinical cohort of carefully followed rheumatoid arthritis patients, a question of interest might be whether pain in joints leads to permanent damage in those joints or, alternatively, what aspects of the disease course are most related to a patient's quality of life.

Often a clinical cohort will aim to begin to follow patients as soon as they are diagnosed with a condition. This would create an ‘inception cohort’. For some diseases, it may be quite straightforward to contact patients at this point. For example, this is often the case with cancer diagnoses. However, in other cases, such as rheumatological diagnoses, it may be much more difficult. Many cohorts are based on patients referred to tertiary treatment centres and these patients may come for treatment at any stage of disease. Nevertheless, important information on disease course can derive from non-inception cohorts. In addition, an inception sub-cohort can sometimes be identified from a larger cohort.

## Case-control and related studies

The collection of cohort data is time-consuming and expensive. This is particularly true in the case of epidemiological investigations of rare diseases and, therefore, a very important study design in epidemiology is the case-control study, which might equally be termed a case-noncase study. This involves the selection of a random sample of incident cases of the study disease in a defined population during a specified case accession period. Corresponding comparison individuals (the noncases or controls) are randomly selected from those members of the same population, or a specified subset of it, who are disease-free during the case accession period. Information on the values of explanatory variables during the time period prior to case or control ascertainment is obtained at the time of ascertainment. These retrospective data are usually subject to more error in measurement than the prospective data of a cohort study; however, a case-control study can be completed more quickly. The case-control design facilitates comparisons of disease rates in different subsets of the study population but, since the numbers of cases and controls sampled are fixed by the design, it cannot provide an estimate of the actual disease rates. Case-control designs vary in the degree of matching of cases to controls with respect to disease risk factors other than the exposure under study.

Most frequently, the analysis of case-control studies is based on logistic regression with a binary outcome, Y, specifying disease status. If case-control data are analysed using a logistic regression model, then although the estimate of the parameter a has no practical value because disease incidence can not be estimated, the estimation of odds ratios, through the b parameters, can proceed in the usual fashion.

More recently, variations on the cohort and case-control designs are being used. Some of these designs involve case-control sampling, perhaps matched on time, from a prospective cohort. In such a study, some data on risk factors may not be collected until an individual is sampled. For example, if blood samples are collected from all individuals in the cohort, blood tests or genetic typing need only to be done on sampled individuals. When matching on time, individuals may be sampled as controls at one time point but become a case at a later time point. This is often referred to as a nested case-control study.

A second type of study involves specifying a cohort within which events of interest, such as disease diagnoses, may occur. Then a random sample of cohort members is selected, and data from these individuals are acquired. This data collection may be done prospectively or retrospectively, depending on the questions of interest and the availability of data on this ‘subcohort’. This subcohort provides the controls in the analysis, but may include individuals who develop the disease of interest at some point. Also collected are data on all, or a random sample of, remaining individuals in the cohort who develop the disease. This type of study is known as a case-subcohort study or simply as a case-cohort study.

Some care is required in choosing and carrying out an appropriate analysis of data collected during a nested case-control design, case-cohort or other similar studies. However, regression models remain the basis of the analysis.

A case-control or similar design can also address clinical questions related to patients with particular medical conditions. No additional methodological issues arise.

## Diagnostic studies

To consider designs to look at the performance of a diagnostic test, Table 2 provides some illustrative numbers to highlight the main features. Assume that 200 individuals are sampled and that 40 of these are found to have a disease D through‘gold-standard’ testing or observation which is assumed to be completely accurate. These 200 individuals are tested with a diagnostic test T of interest and 32 of the 40 diseased and 40 of the 160 non-diseased individuals test positive.

Two key calculations would be an estimate of the sensitivity, which is the probability that a diseased individual tests positive, and the specificity which is the probability that a non-diseased individual tests negative. From Table 2, the estimate of sensitivity is 32/40 = 80% and that of specificity is 120/160 = 75%.

While these two probabilities specify aspects of the diagnostic test's performance, the test's usefulness is also influenced by the prevalence of the disease in the population on which it is used. Two quantities that reflect this are termed the predictive value of a positive test, the probability that an individual with a positive test has the disease, and the predictive value of a negative test, the probability that an individual with a negative test does not have the disease. Estimates of these quantities from Table 2 would be 32/72 = 44.4% and 120/128 = 93.8% respectively. It can be seen that high sensitivity and specificity values do not necessarily translate into uniformly good predictive performance. It follows that the value of the test may differ depending on the population of interest and the relative importance of false positive and false negative tests.

A study to assess a diagnostic test will generally aim to estimate sensitivity and specificity to a required accuracy. Thus, unlike the scenario of Table 2 where it was assumed that 200 individuals from the population of interest are sampled, it would generally be better, if possible, to recruit separate samples of diseased and non-diseased individuals of a required size.

A study of 100 diseased individuals and 100 non-diseased individuals from the population reflected in Table 2 might generate results as given in Table 3.

Based on a simple logistic regression model, the estimated specificity from Table 2 would be 75% with a 95% confidence interval (CI) of (67.7%, 81.1%). From Table 3 it would be 75% with CI (65.6%, 82.5%). Similarly, the estimated sensitivity from Table 2 would be 80% with CI (64.8%, 89.7%), whereas from Table 3 it would be 80% with CI (71.0%, 86.7%). The common total number of individuals in Table 2, Table 3 is simply for illustration and in general the numbers of diseased and non-diseased individuals can be specified independently. However, it can be seen that while Table 3 generates a slightly wider CI for specificity (because the number of non-diseased individuals is slightly smaller) there is a considerable narrowing of the CI for sensitivity.

The design of a diagnostic study should therefore be driven by a desired accuracy for the estimated sensitivity and specificity. Given an expected prevalence, the corresponding predictive values of positive and negative tests can then be calculated. The variation in predictive performance with prevalence can then be seen or predictive performance can be assessed for an individual from a population for which the prevalence is determined from other sources.

The above discussion has presumed that the diagnostic test of interest provides a simple yes/no test result. An alternative is that the diagnostic test provides a continuous measurement, say X, and a value c is to be chosen such that, if X is greater than c, the test will be deemed positive and otherwise negative. Then the sensitivity and specificity vary with the choice of c where as c increases, specificity increases but sensitivity decreases. A plot of sensitivity versus specificity as c varies is termed, for historical reasons, a receiver-operator-characteristic (ROC) curve and the performance of the diagnostic measure is sometimes defined in terms of the shape of this curve. From a design perspective, the key factor will still be the accuracy of sensitivity and specificity estimates.

## Funding

VTF's work was supported by Medical Research Council (UK) funding U105261167.

## Figures and Tables

**Table 1:** Observation numbers in two basic design structures

		I		II	
T_1_	T_2_	T_1_	T_2_
S_1_	Plate 1	50	50	100	0
Plate 2	50	50	0	100
S_2_	Plate 3	50	50	100	0
Plate 4	50	50	0	100

**Table 2:** Illustrative example of a study of diagnostic testing

	D+	D−	
T+	32	40	72
T−	8	120	128
	40	160	200

**Table 3:** Second example of a study of diagnostic testing

	D+	D−	
T+	80	22	102
T−	20	78	98
	100	100	200
